# Adjunctive Atypical Antipsychotic Treatment for Major Depressive Disorder: A Meta-Analysis of Depression, Quality of Life, and Safety Outcomes

**DOI:** 10.1371/journal.pmed.1001403

**Published:** 2013-03-12

**Authors:** Glen I. Spielmans, Margit I. Berman, Eftihia Linardatos, Nicholas Z. Rosenlicht, Angela Perry, Alexander C. Tsai

**Affiliations:** 1 Department of Psychology, Metropolitan State University, St. Paul, Minnesota, United States of America; 2 Department of Psychiatry, Dartmouth Medical School, Lebanon, New Hampshire, United States of America; 3 Department of Psychiatry, University of California at San Francisco, San Francisco, California, United States of America; 4 San Francisco Veterans Affairs Medical Center, San Francisco, California, United States of America; 5 Center for Global Health, Massachusetts General Hospital, Boston, Massachusetts, United States of America; 6 Department of Psychiatry, Chester M. Pierce, MD Division of Global Psychiatry, Massachusetts General Hospital, Boston, Massachusetts, United States of America; 7 Harvard Medical School, Boston, Massachusetts, United States of America; University of Western Sydney, Australia

## Abstract

**Background:**

Atypical antipsychotic medications are widely prescribed for the adjunctive treatment of depression, yet their total risk–benefit profile is not well understood. We thus conducted a systematic review of the efficacy and safety profiles of atypical antipsychotic medications used for the adjunctive treatment of depression.

**Methods and Findings:**

We included randomized trials comparing adjunctive antipsychotic medication to placebo for treatment-resistant depression in adults. Our literature search (conducted in December 2011 and updated on December 14, 2012) identified 14 short-term trials of aripiprazole, olanzapine/fluoxetine combination (OFC), quetiapine, and risperidone. When possible, we supplemented published literature with data from manufacturers' clinical trial registries and US Food and Drug Administration New Drug Applications. Study duration ranged from 4 to 12 wk. All four drugs had statistically significant effects on remission, as follows: aripiprazole (odds ratio [OR], 2.01; 95% CI, 1.48–2.73), OFC (OR, 1.42; 95% CI, 1.01–2.0), quetiapine (OR, 1.79; 95% CI, 1.33–2.42), and risperidone (OR, 2.37; 95% CI, 1.31–4.30).

The number needed to treat (NNT) was 19 for OFC and nine for each other drug. All drugs with the exception of OFC also had statistically significant effects on response rates, as follows: aripiprazole (OR, 2.07; 95% CI, 1.58–2.72; NNT, 7), OFC (OR, 1.30, 95% CI, 0.87–1.93), quetiapine (OR, 1.53, 95% CI, 1.17–2.0; NNT, 10), and risperidone (OR, 1.83, 95% CI, 1.16–2.88; NNT, 8). All four drugs showed statistically significant effects on clinician-rated depression severity measures (Hedges' *g* ranged from 0.26 to 0.48; mean difference of 2.69 points on the Montgomery–Asberg Depression Rating Scale across drugs). On measures of functioning and quality of life, these medications produced either no benefit or a very small benefit, except for risperidone, which had a small-to-moderate effect on quality of life (*g* = 0.49).

Treatment was linked to several adverse events, including akathisia (aripiprazole), sedation (quetiapine, OFC, and aripiprazole), abnormal metabolic laboratory results (quetiapine and OFC), and weight gain (all four drugs, especially OFC). Shortcomings in study design and data reporting, as well as use of post hoc analyses, may have inflated the apparent benefits of treatment and reduced the apparent incidence of adverse events.

**Conclusions:**

Atypical antipsychotic medications for the adjunctive treatment of depression are efficacious in reducing observer-rated depressive symptoms, but clinicians should interpret these findings cautiously in light of (1) the small-to-moderate-sized benefits, (2) the lack of benefit with regards to quality of life or functional impairment, and (3) the abundant evidence of potential treatment-related harm.

*Please see later in the article for the Editors' Summary*

## Abstract 2

In a systematic review and meta-analysis, Glen Spielmans and colleagues find that adjunctive atypical antipsychotic medications are associated with small-to-moderate improvements in depressive symptoms in patients with depression, but there is little evidence for improvement on measures of quality of life, and these medications are linked to adverse events such as weight gain.

## Editors' Summary

**Background:**

Everyone feels miserable occasionally. But for people who are clinically depressed, feelings of sadness and hopelessness and physical symptoms such as sleeping badly can last for months or years and can make them feel life is no longer worth living. Depression affects one in six people at some time during their life. Clinicians diagnose depression by asking their patients a series of questions about their feelings and symptoms. The answer to each question is given a score, and the total score from the questionnaire (“depression rating scale”) indicates the severity of depression. Treatment of depression often involves talking treatments (psychotherapy) such as cognitive behavioral therapy, which helps people change negative ways of thinking and behaving and antidepressant drugs, most commonly “selective serotonin reuptake inhibitors” such as fluoxetine and paroxetine.

**Why Was This Study Done?:**

Atypical antipsychotic medications (for example, aripiprazole, olanzapine/fluoxetine combination [OFC], quetiapine, and risperidone) are also widely prescribed for the treatment of depression. These drugs, which were developed to treat mental illnesses that are characterized by a loss of contact with reality, are used as adjunctive therapy for depression. That is, they are used in addition to antidepressant drugs. Clinicians wrote nearly four million prescriptions for adjunctive treatment of depression with atypical antipsychotic medications in 2007–2008 in the US alone. However, it is not known whether the benefits of using these drugs to treat depression outweigh their side effects, which include weight gain, sedation, and akathisia (a feeling of inner restlessness resulting in an urge to move, which may or may not be accompanied by increased movement). Here, the researchers undertake a systematic review and meta-analysis of the efficacy and safety profiles of atypical antipsychotic medications used for the adjunctive treatment of depression. A systematic review uses predefined criteria to identify all the research on a given topic; a meta-analysis is a statistical approach that combines the results of several studies.

**What Did the Researchers Do and Find?:**

The researchers identified 14 short-term randomized controlled trials (duration 4–12 weeks) that compared adjunctive antipsychotic medications (aripiprazole, OFC, quetiapine, or risperidone) to placebo (dummy drug) in the treatment of depression that had not responded to antidepressant medication alone. All four drugs had statistically significant effects (effects unlikely to have happened by chance) on remission, which was most commonly defined as a score of less than eight at the study end point on the Montgomery–Asberg Depression Rating Scale. The researchers calculated the number of patients that would have to be treated for one patient to achieve remission (number needed to treat, or NNT). For OFC, the NNT was 19; for all the other drugs it was nine. All the drugs except OFC also significantly improved response rates (defined as a 50% improvement in depression rating score). However, the medications provided little or no benefit in terms of functioning and quality of life, except for risperidone, which had a small-to-moderate effect on quality of life. Finally, treatment with atypical antipsychotic medications was linked to several adverse effects, including weight gain (all four drugs) and akathisia (aripiprazole).

**What Do These Findings Mean?:**

These results suggest that atypical antipsychotic medications for the adjunctive treatment of depression are efficacious in reducing observer-rated depressive symptoms. However, clinicians should interpret this conclusion cautiously for several reasons. First, adjunctive treatment with atypical antipsychotics provided only small-to-moderate benefits. Moreover, shortcomings in study design and data reporting methods may have inflated the apparent benefits of treatment and reduced the apparent incidence of adverse events. Second, this study provides little evidence that adjunctive treatment with atypical antipsychotics improves patients' quality of life or reduces their functional impairment. Finally, this study highlights abundant evidence of potential treatment-related harm. This evaluation of the safety and efficacy of adjunctive treatments for clinical depression provides critical insights that should help clinicians better understand the risk–benefit profiles of this approach to the treatment of major depressive disorder.

**Additional Information:**

Please access these websites via the online version of this summary at http://dx.doi.org/10.1371/journal.pmed.1001403.

- The US National Institute of Mental Health provides information on all aspects of depression (in English and Spanish); it has a webpage on mental health medications that includes information about atypical antipsychotics
- The UK National Health Service Choices website also provides detailed information about depression and includes personal stories about depression
- More personal stories about depression are available from healthtalkonline.org
- The UK charity Mind provides information on depression and on antipsychotic drugs; Mind also includes personal stories about depression on its website
- MedlinePlus provides links to other resources about depression (in English and Spanish)
- Healthy Skepticism is an international nonprofit membership association that aims to improve health by reducing harm from misleading health information

## Introduction

Atypical antipsychotic medications are widely used in the treatment of major depressive disorder. In the United States in 2007 and 2008, there were an estimated 3.9 million treatment visits per year in which an antipsychotic medication was prescribed for depression, and nearly all of these (96%) involved prescription of an atypical antipsychotic medication [1]. Although aggregate statistics mask the specific indications for use (i.e., monotherapy versus adjunctive therapy), this represents a substantial increase in antipsychotic treatment of depression over time, as there were just over 2 million such visits annually during 1995 and 1996, of which 405,000 involved prescriptions for atypical antipsychotic medications. These data are also consistent with market reports from industry [2]. Three atypical antipsychotic medications have approval from the US Food and Drug Administration (FDA) as adjunctive therapies in depression for adults, while none are approved for monotherapy. These approvals (and subsequent marketing efforts), along with the volume of prescriptions, suggest that a large number of prescriptions for atypical antipsychotic medications written for the treatment of depression are being used for adjunctive therapy [3]–[5].

The efficacy of adjunctive atypical antipsychotic therapy in reducing depression symptom severity in major depressive disorder is summarized in two previous systematic reviews, but neither comprehensively summarized data on both efficacy and safety [6],[7]. Both reviews analyzed efficacy only in terms of dichotomous response and remission outcomes derived from clinician-rated depression measures and did not assess changes in terms of symptom severity on the underlying continuous rating scales. Safety was only assessed by examining dropout rates due to adverse events; the authors of these meta-analyses [6],[7] and of a relevant narrative review noted that a comprehensive summary of safety data is lacking [8]. A Cochrane review provided a more thorough assessment of both efficacy and safety outcomes but did not include data on important patient-centered efficacy outcomes such as patient-rated depression, functional impairment, or quality of life [9]. The Cochrane review assessed the frequency of several relevant adverse events, but some critical adverse events of interest, such as elevated cholesterol or triglyceride levels, were not included. Further, and most importantly, effect size estimates presented in these reviews may have been inflated because the authors did not summarize unpublished data, such as those from FDA New Drug Applications (NDAs) or manufacturers' clinical trial registries [10]–[13]. Given the importance of functional status, quality of life, and drug-related side effects to the overall assessment of well-being and recovery from depressive mood episodes [14]–[16], we conducted this meta-analysis to provide a comprehensive estimate of the efficacy and safety profiles of atypical antipsychotic medications for the adjunctive treatment of major depressive disorder.

## Methods

### Ethical Review

Because this was a study-level systematic review and meta-analysis of trials, and did not involve collection and analysis of any individual-level data, ethical approval was not sought for this study.

### Search Strategy

This systematic review was reported using PRISMA guidelines; the PRISMA checklist is provided as Text S1. To identify both published and unpublished studies for review, we searched Medline, PsycINFO, ClinicalTrials.gov, and the Cochrane Central Register of Controlled Trials using the terms *depression* AND (*aripiprazole* OR *asenapine* OR *clozapine* OR *iloperidone* OR *lurasidone* OR *olanzapine* OR *paliperidone* OR *quetiapine* OR *risperidone* OR *ziprasidone*). Medline search results were restricted to the following article types: clinical trial, controlled clinical trial, or randomized controlled trial. Our literature search was conducted in December 2011 and updated on December 14, 2012. In addition, we searched the American Psychiatric Association Annual Meeting New Research Abstracts for 2001–2010 using each of the generic drug names as a search term, then winnowed the results down to abstracts that appeared to possibly meet the inclusion criteria. We also examined all references in a previously published meta-analysis [6] as well as those contained in each published study obtained through our literature search.

To obtain additional unpublished data, we searched the drug manufacturers' online clinical trial registries as well as FDA NDAs for the atypical antipsychotic medications that have received an indication for the adjunctive treatment of major depressive disorder (aripiprazole, olanzapine-fluoxetine combination [OFC], and quetiapine). For published studies, we supplemented published data with data available in NDAs or clinical trial registry reports whenever such data were available.

### Study Selection

Trials were included if they were acute-phase (i.e., not for relapse prevention or maintenance treatment [17],[18]), placebo-controlled trials in which participants treated with antidepressant medications were randomly assigned to additionally receive an atypical antipsychotic medication or placebo. In order to meet our definition of treatment-resistant depression, participants must have been diagnosed with current major depressive disorder and must have been determined to have had an inadequate response to at least one course of antidepressant medication treatment prior to enrollment in the study. Furthermore, data for at least one outcome measure must have been reported in a manner that allowed calculation of an effect size. No language exclusions were applied.

### Data Extraction

Four study authors (G. I. S., A. P., M. I. B., and E. L.) coded study descriptor data. To establish consistency, all coders first coded the articles reporting outcomes from the aripiprazole studies. Then two study authors (G. I. S. and A. P.) jointly coded the OFC and risperidone articles, and two study authors (M. I. B. and E. L.) jointly coded the quetiapine articles. Disagreements were resolved by consensus. Coders were not blind to the results of the coded studies.

Several descriptor variables were coded for each study. (1) Flexible dosing versus fixed dosing regimen. (2) Dosage range. (3) Mean dosing achieved at end point. (4) Number of participants in each group of trial. (5) Duration of acute-phase treatment (weeks). (6) Number of prior failed trials of antidepressant medications, where the number of failed trials prior to study enrollment (historical) and the number of failed trials during the study (prospective) prior to initiation of the study drug for adjunctive treatment were recorded separately. (7) Procedures employed to evaluate for major depressive disorder (structured interview or otherwise). (8) Use of a structured instrument versus open-ended questioning to elicit adverse events [19],[20] (with the latter assumed if no details were reported). (9) Adverse events scale(s) used to systematically assess for any particular adverse event(s), if any. (10) The criterion used to establish a minimum level of occurrence for adverse events reporting in the trial (e.g., if only those adverse events occurring in at least 5% of participants were reported in the associated journal article, the adverse events reporting threshold was coded as 5%). (11) Extent to which the random-sequence generation procedures were adequate versus inadequate or unclear [21]. Adequate sequence generation procedures included use of a computer program, random number table, coin tossing, randomly drawing envelopes, throwing dice, or similar methods. Merely describing the trial as randomized was considered an unclear method of sequence generation. (12) Whether or not the study eliminated placebo responders prior to randomization. (13) Whether or not persons who had a prior nonresponse to the study drug were excluded. (14) Whether or not the placebo was described as identical to the study drug in terms of at least two of the following three criteria: taste, appearance, and smell [22]. (15) Use of blinded raters, coded as affirmative if the following two conditions were met: (a) it was explicitly stated that blinded raters were used, and (b) it was explicitly stated that different personnel were used to rate efficacy measures and adverse events [23]–[25]. (16) Funding sponsor.

Efficacy and safety outcome data were independently extracted by two authors (G. I. S. and A. P.) and then checked for agreement. Disagreements were resolved by checking the original data source.

### Outcome Measures

Remission was defined variably across studies. We recorded the most stringent definition of remission utilized in each trial while also recognizing that the Montgomery–Asberg Depression Rating Scale (MADRS) [23] was the most commonly used outcome measure in the included trials. One end-point remission measure was selected from each trial according to the following order of priority: MADRS ≤8, then Hamilton Depression Rating Scale (HAM-D) ≤7 [24], then MADRS ≤10. Some trials of OFC defined remission as MADRS ≤8 at two consecutive visits during the study even if these two consecutive visits did not necessarily occur at study end point [25]–[27]. The clinical trial registry reports of these trials also provided the number of participants who met remission criteria at an interim time point but then relapsed. For these studies, we calculated the number of participants in remission as the number of participants who achieved interim remission minus the number of patients who subsequently relapsed.

Response was defined across studies as a 50% improvement from baseline to end point on either the MADRS or HAM-D [28]. When studies provided response rates for both measures, we used the MADRS as the response measure, as it was the most commonly reported measure of response.

We recorded data from any continuous measure of depression, quality of life, or functioning but opted not to analyze single rating scale items from larger scales (e.g., individual MADRS items) separately because they were infrequently reported. When data were reported on both the MADRS and HAM-D, we included data from the MADRS, as it was the most commonly used measure of depressive symptoms. The only continuous self-report measure of depression used in these trials was the Inventory of Depressive Symptomatology Self Report [29]. Continuous measures of quality of life included the Quality of Life Enjoyment and Satisfaction Questionnaire (Q-LES-Q) [30] and the Short Form 36 Health Survey (SF-36) [31]. The only continuous measure of functional impairment employed in these trials was the Sheehan Disability Scale (SDS) [32]. As measures of quality of life and functional impairment varied across studies, we pooled such measures together to create an omnibus effect size for each drug, and across all drugs.

We aggregated conceptually similar adverse events into the following categories. (1) Sedation-related: asthenia, fatigue, lethargy, sedation, somnolence, or feeling tired. (2) Akathisia-related (either self-reported or observer-rated): akathisia or restlessness. (3) Extrapyramidal symptoms (EPS), other than akathisia-related (either self-reported or observer-rated): dyskinesia, dystonia, extrapyramidal disorder, EPS, muscle spasms, muscle twitching, parkinsonism, or tremor. (4) Abnormal metabolic laboratory results: elevated fasting or nonfasting total cholesterol, low-density lipoprotein (LDL) cholesterol, or triglycerides; low high-density lipoprotein (HDL) cholesterol; or elevated fasting or nonfasting glucose, glycated hemoglobin; or hyperglycemia. (5) Elevated prolactin. (6) Edema or peripheral edema. (7) Significant weight gain, defined across various trials as weight gain of ≥7%, ≥10%, or >10% from baseline to end point.

We also coded events that were reported in the categories of pain, psychiatric events, nausea, and infection. However, because no sign of elevated risk was gleaned from these data, these analyses are not reported (data available from authors on request).

### Statistical Analysis

The quality of data reporting varied across studies. For continuous outcomes, effect sizes were computed from means and standard deviations when possible. When these were not provided, effect sizes were computed based on means and *p*-values, or *p*-values only. In some studies, three or more treatment groups were compared, thereby creating a structural dependency that could affect our estimates. For example, two fixed doses (A and B) of an adjunctive atypical antipsychotic medication might be compared to one group that received adjunctive placebo (C), in which case the estimated efficacy of A and B would be defined relative to the same comparison group. To maintain independence, we pooled these comparisons and utilized their average (e.g., the average of A versus C and B versus C).

Each effect size was weighted by its inverse variance in order to provide a pooled effect size estimate that most accurately approached the true population effect size [33]. We calculated odds ratios (ORs) for categorical measures and used Cohen's *d* for continuous measures. We converted continuous effect sizes to Hedges'*g*, which corrects for a small bias in Cohen's *d* [33]. We reported both efficacy and safety data for each drug individually and across drugs. An OR presents a relative measure of treatment effect; to also provide a measure of absolute benefit/harm, we calculated the number needed to treat (NNT) for treatment benefits and the number needed to harm (NNH) for adverse events [34]. The NNT represents the number of participants who would need to be treated with an adjunctive antipsychotic to gain one additional beneficial response over what would have been obtained had all patients received adjunctive placebo. NNH represents the number of patients who would require treatment to generate one additional adverse event relative to placebo. NNT/NNH values were calculated based on the pooled OR rather than from the risk difference in each study, as the risk difference is associated with more between-study heterogeneity than the OR [35]. Conversions from OR to NNT were performed in Visual Rx software [36]. The baseline risk (required for calculating NNT) was estimated by using the pooled rate of events occurring among placebo-treated patients weighted by each study's total sample size. The baseline risk was calculated separately for each drug, so that placebo participants in one drug's trials were not used to calculate baseline risk for a different drug. As in any meta-analysis, our estimates of NNT and NNH generalize only to situations in which patients receive a similar dosage for a similar treatment duration; further, estimated NNH and NNT apply only when generalizing to patients similar to those in the included trials. Because of various study inclusion and exclusion criteria, patients in the placebo groups in our meta-analysis may not be representative of patients seen in some clinical practice settings.

We performed homogeneity analyses using the *Q* statistic. Because the *Q* test of homogeneity often lacks power to detect heterogeneity when the number of trials in a meta-analysis is small, we also calculated the *I* ^2^ statistic [37]. To pool estimates across studies while incorporating potential heterogeneity, we employed a random effects model in all analyses [38]. Confidence intervals for *I* ^2^ were calculated using Method III as described in Higgins and Thompson [39] using a spreadsheet. When performing such calculations in pooled analyses based on only two comparisons when *Q*≤*k*, we added the number 1 to both *Q* and *k* in order to avoid the mathematical problem of dividing by zero; this generally resulted in a slight shrinking of the confidence intervals under these conditions. Unless specified otherwise above, all analyses were performed using Comprehensive Meta-Analysis software [40]. We lacked adequate statistical power to perform subgroup analyses.

We examined the potential existence of publication bias by performing trim and fill analysis for pooled continuous depression outcomes. Trim and fill procedures examine potential asymmetry of effect sizes. Based on the assumption that effects are distributed symmetrically, trim and fill analysis imputes the number and likely effect size of missing studies, then recalculates the pooled analysis with imputed data from missing studies [41].

## Results

### Study Characteristics

**Figure 1:**
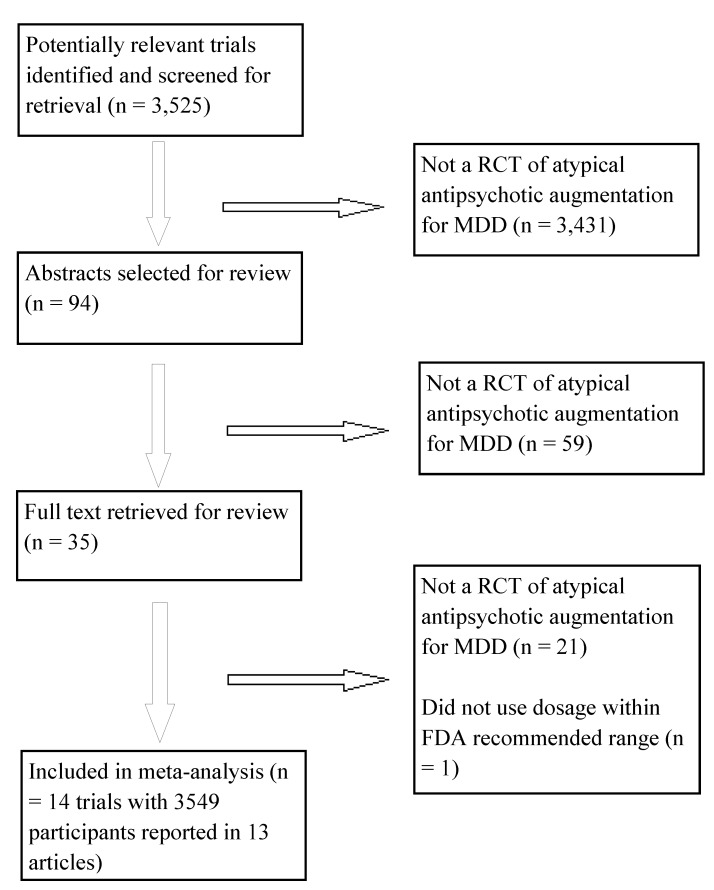
Flowchart of published studies examined for inclusion in meta-analysis. MDD, major depressive disorder; RCT, randomized controlled trial.

The evidence search flow is described in Figure 1. We obtained one controlled trial of aripiprazole that used low doses (2 or 5 mg); we did not include this trial because the starting dose of 2 mg was administered for 30 d prior to participants switching to the dose of 5 mg that falls within the recommended 5–10 mg range set by the FDA [42]. Characteristics of the 14 included studies are provided in Table 1. The definition of treatment-resistant depression differed somewhat across trials. The process by which diagnoses were made was described clearly in six trials, and the number of prior failed trials varied across studies. Only three studies clearly described their random-sequence generation procedures, and only one trial clearly described using clinical raters who were blind to both treatment assignment and participants' reports of adverse events. While most trials used rating scales to assess for EPS and akathisia, and a minority of trials used a measure of sexual functioning, no trial reported using a structured instrument for eliciting a broad range of adverse events. All studies were funded by the study drug manufacturer except for one trial that was funded jointly by the study drug manufacturer and the US National Institute of Mental Health [27].

**Table 1:** Characteristics of included studies.

Study First Author (Year) [Reference]	Antipsychotic	Antidepressant	Daily Dosage at End Point	*N* a	Mean Age (Years)	Percent Female	Duration (Weeks)	Prior Failed Trials	Interview to Establish Diagnosis^b^	Categorical Depression Measures^c^	Supplemental Data Sources^d^	Adverse Events Assessed Systematically	Adverse Events Reporting Threshold	Adequate Sequence Generation?	Established Placebo Resistance?	Prior Drug Nonresponders Excluded?	Placebo Similarity?	Blinded Raters?
Bauer (2009) [81]	Quetiapine	Various	Fixed, 150 or 300 mg	487	45.4	67.6	6	1 historical	MINI	Remission: MADRS ≤8; response: MADRS	CTR, FDA	Akathisia, EPS,sexual functioning	>5% in any group	?	No	Yes	?	?
Berman (2007) [75]	Aripiprazole	Various	Flexible, M = 11.8 mg	353	45.4	62.8	6	1–3 historical, 1 prospective	?	Remission: MADRS ≤10; response: MADRS	CTR, FDA	Akathisia, EPS, sexual functioning	≥5% in any group	Yes	Yes	Yes	?	?
Berman (2009) [82]	Aripiprazole	Various	Flexible, M = 10.7 mg	343	45.3	73.1	6	1–3 historical, 1 prospective	?	Remission: MADRS ≤8; response: MADRS	CTR	Akathisia, EPS, sexual functioning	≥5% in any group	?	Yes	Yes	?	?
Corya (2006) [25]	OFC	Fluoxetine or venlafaxine	Fixed; olanzapine 6 mg/fluoxetine 25 mg, olanzapine 6 mg/fluoxetine 50 mg, olanzapine 12 mg/fluoxetine 25 mg, or olanzapine 12 mg/fluoxetine 50 mg	344	45.7	72.5	12	1 historical, 1 prospective	?	Remission: MADRS ≤8 at two consecutive visits excluding patients who relapsed; response: MADRS	CTR	Akathisia, EPS	≥10% in OFC group	?	No	No	?	?
El-Khalili (2010) [83]	Quetiapine	Various	Fixed, 150 or 300 mg	432	45.5	72.5	6	1 historical	MINI	Remission: MADRS ≤8; response: MADRS	CTR, FDA	Akathisia, EPS, sexual functioning	>5% in any group	Yes	No	No	Yes	?
Keitner (2009) [73]	Risperidone	Various	Flexible, M = 1.6 mg	95	45.2	56.7	4	1 prospective	SCID	Remission: HAM-D ≤7; response: MADRS	None	None	?	?	No	No	?	Mostly^e^
Mahmoud (2007) [44]	Risperidone	Various	Flexible, M = ?, 1 or 2 mg permitted	268	46.1	73.5	6	1 prospective	?	Remission: HAM-D ≤ 7; response: HAM-D	None	None	≥2% in any group	Yes	No	No	?	?
Marcus (2008) [76]	Aripiprazole	Various	Flexible, M = 11.0 mg	369	44.5	66.7	6	1–3 historical, 1 prospective	?	Remission: MADRS ≤10; response: MADRS	CTR, FDA	Akathisia, EPS, sexual functioning	≥5% in any group	?	Yes	Yes	?	?
McIntyre (2007) [84]	Quetiapine	Various	Flexible, M = 182 mg	58	44.5	62.0	8	1 trial	?	Remission: HAM-D ≤7; response: HAM-D	None	None	?^f^	?	No	No	?	?
Reeves (2008) [77]	Risperidone	Various	Flexible, M = 1.17 mg	23	44.0	69.6	8	1 prospective	?	Remission: N/A; response: N/A	None	Akathisia (one item from EPS scale), EPS	≥13% of total participants	?	No	No	?	?
Shelton (2001) [27]	OFC	Fluoxetine	Flexible, mean modal dose = olanzapine 13.5 mg/fluoxetine 52 mg	20	42.0	75	8	2 historical and 1 prospective	?	Remission: MADRS ≤8 at two consecutive visits excluding patients who relapsed; response: MADRS	CTR	Akathisia, EPS	Number of adverse events not reported	?	No	No	?	?
Shelton (2005) [26]	OFC	Fluoxetine or nortriptyline	Flexible, mean modal dose = olanzapine 8.5 mg/fluoxetine 35.6 mg	356	42.0	69.4	8	1 historical, 1 prospective	SCID	Remission: MADRS ≤8 at two consecutive visits excluding patients who relapsed; response: MADRS	CTR	Akathisia, EPS	≥10% of OFC group	?	No	No	?	?
Thase 1 (2007) [85] g	OFC	Fluoxetine	Fixed; olanzapine 6 mg/fluoxetine 50 mg, olanzapine 12 mg/fluoxetine 50 mg, or olanzapine 18 mg/fluoxetine 50 mg	203	44.1	60.2	8	1 historical, 1 prospective	SCID	Remission: MADRS ≤10; response: MADRS	CTR	Akathisia, EPS	≥10% of OFC group	?	No	No	?	?
Thase 2 (2007) [85] g	OFC	Fluoxetine	Fixed; olanzapine 6 mg/fluoxetine 50 mg, olanzapine 12 mg/fluoxetine 50 mg, or olanzapine 18 mg/fluoxetine 50 mg	198	44.9	68.0	8	1 historical, 1 prospective	SCID	Remission: MADRS ≤10; response: MADRS	CTR	Akathisia, EPS	≥10% of OFC group	?	No	No	?	?

a Number of participants included in the intent-to-treat or modified intent-to-treat analysis on the primary depression rating scale in the trial.

b If no interview was explicitly mentioned, then this variable was coded as “?”; MINI, Mini International Psychiatric Interview; SCID, Structured Clinical Interview for DSM-IV.

c Indicates measures used to define remission and/or response in each study.

d “CTR” indicates a clinical trial registry report from the sponsor's online database; “FDA” indicates an FDA statistical review.

e Blinded raters rated outcomes on the MADRS and HAM-D, whereas the study psychiatrist, who also elicited reports of adverse events, rated the Clinical Global Impressions–Severity.

f Adverse events reported by fewer than two of the 29 patients in each group were categorized as “other” in the study's table.

g Data for these two trials were reported separately for some variables but jointly for others.

M, mean.

### Efficacy

**Figure 2:**
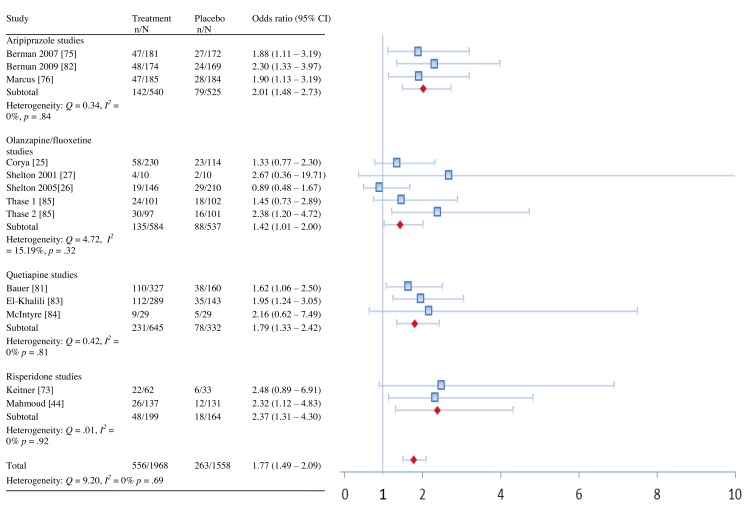
Remission rates by drug and overall.

**Figure 3:**
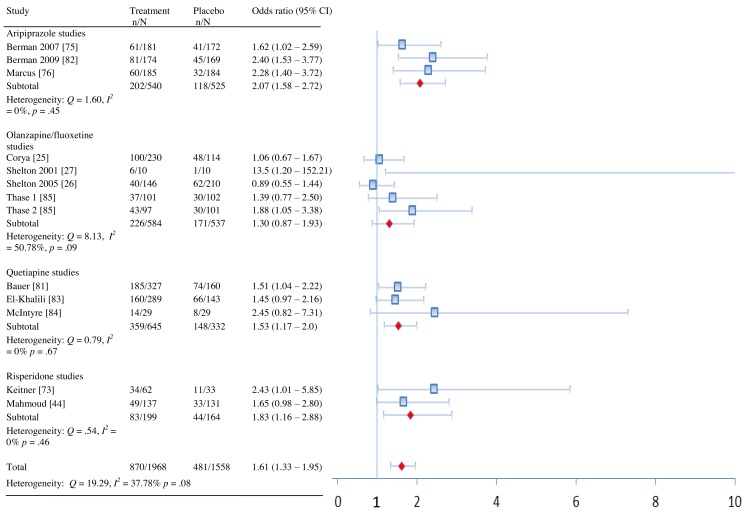
Response rates by drug and overall.

In terms of remission, adjunctive treatment with each antipsychotic was associated with a statistically significant benefit, with ORs ranging from 1.42 to 2.37 (Table 2). ORs for response were also statistically significant for aripiprazole, quetiapine, and risperidone—but not for OFC (Table 2). The NNT for remission was nine for aripiprazole, quetiapine, and risperidone but was a substantially higher 19 for OFC (Table 2). NNTs for response were seven (aripiprazole), eight (risperidone), and ten (quetiapine). Pooled ORs are displayed visually in Figures 2 and 3 [43]. Among participants who achieved remission during treatment, participants assigned to OFC were less likely to remain in remission than participants assigned to placebo. Only two of 56 placebo participants relapsed, compared to 18 relapses among 99 participants on OFC (OR, 0.27; 95% CI, 0.08–0.90).

**Table 2:** Summary of dichotomous efficacy and safety measures.

Comparison	Outcome	*k*	OR (95% CI)^a^	*Q*	*I* ^2^ (95% CI)	*p*(*Q*)	NNT/NNH (95% CI)
**All combined**	Remission	13	1.77 (1.49–2.09)	9.20	0% (0%–43.38%)	0.69	10 (8–15)
	Response	13	1.61 (1.33–1.95)	19.29	37.78% (0%–67.76%)	0.08	9 (7–16)
**Aripiprazole**	Remission	3	2.01 (1.48–2.73)	0.34	0% (0%–38.81%)	0.84	9 (6–18)
	Response	3	2.07 (1.58–2.72)	1.60	0% (0%–87.0%)	0.45	7 (5–12)
	Akathisia	3	7.47 (5.07–11.0)	1.63	0% (0%–87.24%)	0.44	4 (3–6)
	Sedation	3	2.56 (1.63–4.03)	0.68	0% (0%–69.41%)	0.71	14 (8–33)
	Weight gain ≥7%	3	5.91 (2.14–16.29)	0.57	0% (0%–63.50%)	0.75	29 (10–119)
**OFC**	Remission	5	1.42 (1.01–2.0)	4.72	15.19% (0%–82.38%)	0.32	19 (9–713)
	Response	5	1.30 (0.87–1.93)	8.13	50.78% (0%–81.95%)	0.09	17 (NNH 34; NNT 7)^b^
	Weight gain ≥10%	4	16.28 (7.02–37.76)	0.88	0% (0%–47.80%)	0.83	9 (5–20)
	Elevated metabolic lab results	4	4.46 (2.07–9.58)	4.50	33.38% (0%–76.44%)	0.21	10 (5–29)
	Sedation^c^	3	2.87 (1.64–5.03)	7.83	74.45% (0%–92.32%)	0.02	5 (3–12)
	Edema^d^	3	13.19 (5.46–31.89)	0.24	0% (0%–13.32%)	0.89	7 (4–16)
	Elevated prolactin	4	4.30 (2.36–7.83)	4.91	38.84% (0%–79.16%)	0.18	6 (4–11)
	Akathisia	4	1.48 (0.96–2.30)	3.17	5.36% (0%–85.51%)	0.37	28 (NNH 11; NNT 321)^b^
**Quetiapine**	Remission	3	1.79 (1.33–2.42)	0.42	0% (0%–50.47%)	0.81	9 (6–19)
	Response	3	1.53 (1.17–2.0)	0.79	0% (0%–73.67%)	0.67	10 (6–26)
	Sedation	3	8.36 (5.83–11.98)	1.73	0% (0%–87.98%)	0.42	3 (2–3)
	Elevated metabolic lab results	2	2.45 (1.80–3.34)	0.40	0% (0%–85.14%)	0.53	6 (4–9)
	Weight gain ≥7%	3	2.86 (1.11–7.37)	0.97	0% (0%–78.55%)	0.62	37 (12–594)
**Risperidone**	Remission	2	2.37 (1.31–4.30)	0.01	0% (0%–79.40%)	0.92	9 (5–35)
	Response	2	1.83 (1.16–2.88)	0.54	0% (0%–86.49%)	0.46	8 (5–33)

Measures of response and remission are reported for each treatment. Adverse events measures are reported for events that reached a statistical threshold of *p*<0.10 in terms of OR. For further description of the data underlying the adverse events effect sizes, see Table 4.

a Trials with no events in either study arm are not included in summary OR calculations.

b The 95% confidence interval included the possibility of both treatment-related benefit and treatment-related harm.

c Because the total number of events in the OFC group was higher than the sample size of the group in Shelton et al [27], an effect size could not be calculated, and it was thus not factored into the overall effect size estimate for sedation. Given the very small sample of the study, this makes virtually no difference in the overall effect size estimate.

d The four trials in which edema was reported for OFC participants had an average rate of 18.32%. Edema was not listed as an adverse event in Shelton et al [26] for any participants in either the OFC or placebo group. As these data did not fit with the other OFC trials, we excluded this study from the calculation of the risk for placebo participants.

Pooled effect sizes for continuous outcomes are provided in Table 3. Adjunctive aripiprazole, quetiapine, OFC, and risperidone were all more efficacious than adjunctive placebo based on clinician-rated measures of depression severity (MADRS/HAM-D). Effect sizes were as follows: aripiprazole: *g* = 0.35 (95% CI, 0.23–0.48); OFC: *g* = 0.26 (95% CI, 0.04–0.45); quetiapine: *g* = 0.40 (95% CI, 0.26–0.53); and risperidone: *g* = 0.48 (95% CI, 0.22–0.73). The effects of risperidone may have been exaggerated by the reliance on post hoc analysis rather than a priori analysis in the largest study of the drug, as the effect of the drug was greater at 6 wk (*g* = 0.46) than at the prespecified primary end point of 4 wk (*g* = 0.32) [44]. According to convention, these effect sizes would be considered “small” or “small to moderate” in magnitude [45]. Effect sizes on depression severity measures did not differ significantly between drugs (*Q*_B_ = 1.93, *p* = 0.59), though there was limited power to detect such differences. The pooled difference in mean change on the MADRS in the 11 trials that reported such data was 2.69. In these 11 trials, the mean effect size was *g* = 0.31, which differed only slightly from the overall mean effect size when including both the HAM-D and MADRS; thus, the 11 trials reporting MADRS mean change data seem representative of the entire sample of included trials. Only the trials of adjunctive aripiprazole reported self-reported depression symptom severity, yielding a very small effect size of *g* = 0.15. The effects observed on the Clinical Global Impressions–Severity Scale were either small or small-to-moderate, with the exception of risperidone, for which a moderate effect was generated.

**Table 3:** Effect sizes and heterogeneity of effect sizes on continuous measures.

Drug	Outcome Type	Outcome	Study First Author (Year) [Reference]	*g_+_* (95% CI for Totals)	Raw Units (95% CI for Totals)	*p*(*g_+_*)	*Q*	*I* ^2^ (95% CI)	*p*(*Q*)
**Aripiprazole**	Depression	MADRS	Berman (2007) [75]	0.35 (0.33)^a^	3.01 (3.01)				
			Berman (2009) [82]	0.38	3.73				
			Marcus (2008) [76]	0.32 (0.26)^a^	2.84 (2.52)				
			**Total**	0.35 (0.23, 0.48) (0.33 [0.20, 0.47])^a^	3.15 (2.07, 4.23) (3.14 [1.87, 4.41])^a^	<0.001 (<0.001)^a^	0.18 (0.53)^a^	0% (0%–0%) (0% [0%–60.75%])^a^	0.92 (0.77)^a^
		IDS-SR	Berman (2007) [75]	0.17					
			Berman (2009) [82]	0.13					
			Marcus (2008) [76]	0.14					
			**Total**	0.15 (0.03, 0.27)		0.02	0.08	0% (0%–0%)	0.96
	QoL/functioning	SDS	Berman (2007) [75]	0.19 (0.10)^a^					
			Berman (2009) [82]	0.16					
			Marcus (2008) [76]	0.25 (0.10)^a^					
			Total	0.20 (0.08, 0.33) (0.12 [−0.02, 0.26])^a^		0.001 (0.08)^a^	0.37 (0.17)^a^	0% (0%–43.78%) (0% [0%–0%])^a^	0.83 (0.92)^a^
		Q-LES-Q	Berman (2007) [75]	0.16					
			Berman (2009) [82]	0.26					
			Marcus (2008) [76]	0.28					
			**Total**	0.23 (0.11, 0.36)		<0.001	0.64	0% (0%–67.50%)	0.73
			**Total QoL/functioning**	0.22 (0.09, 0.34)		0.001	0.33	0% (0%–36.96%)	0.85
	Global improvement	CGI-S	Berman (2007) [75]	0.37					
			Berman (2009) [82]	0.31					
			Marcus (2008) [76]	0.46					
			**Total**	0.38 (0.26, 0.50)		<0.001	1.05	0% (0%–80.19%)	0.59
	Metabolic parameters	Weight gain (kg)	Berman (2007) [75]		1.67				
			Berman (2009) [82]		0.4				
			Marcus (2008) [76]		1.05				
			**Total**		1.05 (0.35, 1.74)	0.003	12.03	83.38% (49.64%–94.51%)	0.002
	Sexual functioning	SFI overall satisfaction	Berman (2007) [75]	?					
			Berman (2009) [82]	0.25					
			Marcus (2008) [76]	?					
			**Total**	?^b^					
**OFC**	Depression	MADRS	Corya (2006) [25]	0.15	1.35				
			Shelton (2001) [27]	1.04	12.40				
			Shelton (2005) [26]	0.08	0.65				
			Thase 1 (2007) [85]	0.14	1.40				
			Thase 2 (2007) [85]	0.57	5.60				
			**Total**	0.26 (0.04, 0.45)	2.57 (0.33, 4.81)	0.02	11.37	64.83% (7.70%–86.59%)	0.02
	QoL	SF-36 MCS	Corya (2006) [25]	ns^c^					
			Shelton (2001) [27]	0.50					
			Shelton (2005) [26]	−0.10					
			Thase (2007) [85]	0.13					
			**Total**	0.04 (−0.17, 0.25)		0.72	3.13	36.18% (0%–79.57%)	0.21
		SF-36 PCS	Corya (2006) [25]	ns^c^					
			Shelton (2001) [27]	−0.06					
			Shelton (2005) [26]	−0.13					
			Thase (2007) [85]	0.19					
			**Total**	0.03 (−0.23, 0.30)		0.82	4.57	56.25% (0%–87.52%)	0.10
			**Total QoL**	0.04 (−0.19, 0.26)		0.74	3.46	42.20% (0%–82.51%)	0.18
	Global improvement	CGI-S	Corya (2006) [25]	0.12					
			Shelton (2001) [27]	0.31					
			Shelton (2005) [26]	0.27					
			Thase 1 (2007) [85]	0.08					
			Thase 2 (2007) [85]	0.32					
			**Total**	0.20 (0.08, 0.32)		0.001	2.35	0% (0%–64.60%)	0.67
	Prolactin	Prolactin (ng/ml)	Shelton (2005) [26]	?^d^					
			Thase (2007) [85]	0.27	2.50				
	Metabolic parameters	Weight gain (kg)	Corya (2006) [25]		3.98				
			Shelton (2001) [27]		5.79				
			Shelton (2005) [26]		3.88				
			Thase (2007) [85]		4.5				
			**Total**		4.20 (3.79, 4.61)	<0.001	3.33	9.80% (0%–86.21%)	0.34
		Cholesterol (total nonfasting, mg/dl)	Corya (2006) [25]	0.28	7.59				
			Shelton (2005) [26]	?^e^	?^e^				
			Thase (2007) [85]	0.43	14.3				
			**Total**	0.37 (0.21, 0.54)	10.85 (4.28, 17.43)	<0.001	1.25	19.88% (0%–90.75%)	0.26
		Triglycerides (mg/dl)	Thase (2007) [85]	0.22	23.90				
			**Total**	0.22 (0.02, 0.41)	23.90 (2.39, 45.41)	0.03	N/A	N/A	N/A
**Quetiapine**	Depression	MADRS	Bauer (2009) [81]	0.41	2.89				
			El-Khalili (2010) [83]	0.34	2.45				
		HAM-D	McIntyre (2007) [84]	0.71	5.70				
			**Total**	0.40 (0.26, 0.53)^f^	2.68^f^	<0.001	1.69	0% (0%–87.69%)	0.43
	QoL	Q-LES-Q	Bauer (2009) [81]	0.10					
			El-Khalili (2010) [83]	−0.02					
			**Total**	0.04 (−0.09, 0.18)		0.53	0.68	0% (0%–87.62%)	0.41
	Global Improvement	CGI-S	Bauer (2009) [81]	0.41					
			El-Khalili (2010) [83]	?^g^					
			McIntyre (2007) [84]	0.69					
			**Total**	0.44 (0.26, 0.62)		<0.001	0.99	0% (0%–89.55%)	0.32
	Prolactin	Prolactin (ng/ml)	Bauer (2009) [81]	0.03	0.44				
		Prolactin (mIU/L)	El-Khalili (2010) [83]	−0.08	−12.12				
			**Total**	−0.02 (−0.16, 0.12)		0.77	0.61	0% (0%–87.08%)	0.43
	Metabolic parameters	HDL cholesterol (mg/dl)	Bauer (2009) [81]	−0.11	−1.0				
			El-Khalili (2010) [83]	−0.21	−1.82	0.02	0.43	0% (0%–85.45%)	0.43
			**Total**	−0.16 (−0.29, −0.02)	−1.38 (−2.58, −0.18)				
		LDL cholesterol (mg/dl)	Bauer (2009) [81]	0.17	4.75				
			El-Khalili (2010) [83]	0.04	0.77				
			**Total**	0.11 (−0.03, 0.24)	2.86 (−1.03, 6.76)	0.12	0.91	0% (0%–89.11%)	0.34
		Total cholesterol (mg/dl)	Bauer (2009) [81]	0.21	6.51				
			El-Khalili (2010) [83]	0.18	4.83				
			**Total**	0.19 (0.06, 0.33)	5.68 (1.57, 9.79)	0.01	0.07	0% (0–80.47%)	0.80
		Triglycerides	Bauer (2009) [81]	0.27	19.65				
		(mg/dl)	El-Khalili (2010) [83]	0.36	38.09				
			**Total**	0.31 (0.17, 0.45)	26.90 (9.24–44.57)	<0.001	0.38	0% (0%–84.93%)	0.54
		Weight gain (kg)	Bauer (2009) [81]		0.95				
			El-Khalili (2010) [83]		0.89				
			McIntyre (2007) [84]		2.65				
			**Total**		0.94 (0.62, 1.26)	<0.001	1.03	0% (0%–79.80%)	0.60
	Sexual functioning	CSFQ	Bauer (2009) [81]	0.01					
			El-Khalili (2010) [83]	?^h^					
			**Total**	?					
**Risperidone**	Depression	MADRS	Keitner (2009) [73]	ns^i^	?				
		HAM-D	Mahmoud (2007) [44]	0.46 (0.32)^j^	2.80 (1.90)^j^				
		MADRS	Reeves	0.60	7.11				
			**Total**	0.48 (0.22, 0.73) (0.34 [0.11, 0.58])^j^^,^ k		<0.001 (0.004)	0.09 (0.42)	0% (0%–80.91%) (0% [0%–85.35%])	0.76 (0.52)
	QoL/functioning	Q-LES-Q	Keitner (2009) [73]	0.54					
			Mahmoud (2007) [44]	0.39^l^					
			**Total**	0.43 (0.20, 0.66)^l^		<0.001	0.33	0% (0%–84.36%)	0.56
		SDS	Mahmoud (2007) [44]	0.57^l^					
			**Total**	0.57 (0.28, 0.85)^l^		<0.001	N/A	N/A	N/A
			**Total QoL/functioning**	0.49 (0.26, 0.73)^l^		<0.001	0.05	0% (0%–80.19%)	0.82
	Global improvement	CGI-S	Keitner (2009) [73]	0.44					
			Mahmoud (2007) [44]	0.72					
			Reeves (2008) [77]	0.78					
			**Total**	0.64 (0.42, 0.87)		<0.001	1.29	0% (0%–83.87%)	0.53
	Prolactin	Prolactin (ng/ml)	Keitner (2009) [73] m						
			Mahmoud (2007) [44] m						
			Reeves (2008) [77]	0.80	38.85				
			**Total**	0.80 (−0.19, 1.80)	38.85 (−5.67, 83.37)	0.11	N/A	N/A	N/A
	Weight gain	Weight gain (kg)	Keitner (2009) [73]		1.81				
			Mahmoud (2007) [44]		1.13				
			Reeves (2008) [77]		−0.45				
			**Total**		1.26	<0.001	3.51	42.95% (0%–82.87%)	0.17
**Summary**		MADRS/HAM-D		0.34 (0.25, 0.43)	2.69 (1.82, 3.54)^n^	<0.001	19.44	38.26% (0%–67.99%)	0.08
		QoL/functioning^o^		0.17 (0.06, 0.28)		0.003	18.25	50.68% (0%–76.05%)	0.03

a The FDA statistical review noted that many participants violated study protocol, often as a result of taking non-allowed medications. The numbers in parentheses represent data from only participants who did not violate the study protocol.

b As data were not presented clearly for two of three trials, we opted to not treat the single study with clearly reported data as representative of the three aripiprazole studies; thus, we provide no summary effect size calculation for sexual functioning.

c “ns” indicates no statistically significant difference versus placebo; data were not reported in a more detailed manner.

d OFC had a greater increase in prolactin versus fluoxetine (0.31 nmol/l, *p*<0.001) and versus nortriptyline (0.37 nmol/l, *p*<0.001). Given the inexact *p*-values, we did not calculate an effect size.

e OFC had a greater increase in mean total nonfasting cholesterol versus fluoxetine (0.30 mmol/l, *p*<0.001) and nortriptyline (0.33 mmol/l, *p* = 0.004). Given the inexact *p*-value of the OFC versus fluoxetine comparison, we did not calculate an effect size.

f Hedges' *g* includes effects from all three trials, whereas the raw unit analysis includes only change on the MADRS, which was not used in the McIntyre et al. [84] study.

g It was reported that 150 mg of quetiapine was superior to placebo, with an associated *p*-value of 0.095, from which a *d* = 0.20 was calculated. For the 300-mg dose, the *p*-value was reported as <0.05, so it was not possible to calculate an exact effect size, as a standard deviation for this measure was not provided.

h There were “no apparent differences among the treatment groups” according to the El-Khalili et al. [83] clinical trial registry report. Given the lack of clarity regarding sexual functioning data, we provide no summary effect size calculation for sexual functioning.

i “ns” indicates no statistically significant difference versus placebo; data were not reported in a more detailed manner.

j The primary efficacy end point in the Mahmoud et al. [44] study was 4 wk, so data in parentheses indicate data from this a priori end point rather than from the 6-wk end point that was emphasized much more heavily in the study publication.

k Hedges' *g* pools data from Mahmoud et al. [44] and Reeves et al. [77]. We provide no summary estimate of mean differences on the MADRS, as only the small Reeves et al. [77] study reported these data.

l Mahmoud et al.'s primary end point was 4 wk [44], but these data are presented at 6 wk. These results may be inflated relative to the primary end point, given that the effect favoring risperidone on the HAM-D was smaller at week 4 than at week 6.

m Prolactin levels were apparently not measured in these trials.

n Pooled raw units are for MADRS only.

o These data are pooled across the SDS, Q-LES-Q, SF-36 Mental Component Summary, and SF-36 Physical Component Summary.

CGI-S, Clinical Global Impressions–Severity; CSFQ, Changes in Sexual Function Questionnaire; IDS-SR, Inventory of Depressive Symptomatology–Self Report; QoL, quality of life; SF-36 MCS, SF-36 Mental Component Summary; SF-36 PCS, SF-36 Physical Component Summary; SFI, Sexual Function Inventory.

With regards to quality of life and functioning, adjunctive quetiapine, aripiprazole, and OFC produced effect sizes that were either not statistically significant or small and clinically negligible in magnitude. Adjunctive risperidone was more efficacious than adjunctive placebo on quality of life/functioning, with a small-to-moderate effect size. The pooled effect across quality of life/functioning measures varied significantly across treatments (*Q*_B_ = 6.88, *p* = 0.003), with risperidone (*g* = 0.49) yielding a higher effect than the other three drugs combined (*g* = 0.11), which did not differ significantly from each other (*Q*_B_ = 4.02, *p* = 0.13). However, the effect of aripiprazole on quality of life/functioning was small (*g* = 0.22) and statistically significant (*p* = 0.001), whereas the effects of OFC (*g* = 0.04, *p* = 0.74), and quetiapine (*g* = 0.05, *p* = 0.53) were both not statistically significant and of quite small magnitude. The effect of aripiprazole on quality of life/functioning should be interpreted with caution, as the effect for the drug on the SDS was very small and no longer statistically significant when patients who violated study protocol were excluded from analysis (*g* = 0.12, *p* = 0.08). Similarly, the effect of risperidone on quality of life/functioning should be interpreted tentatively since it is largely driven by post hoc analyses.

### Adverse Events

Atypical antipsychotic medications differed substantially in their reported adverse event profiles. Table 2 reports adverse events that showed increased risk (*p*≤0.10). A more detailed listing of adverse events and pooled ORs for each event category are provided in Table 4.

**Table 4:** Adverse events individually and by category.

Drug	Study First Author (Year) [Reference]	Event	Events/*N* on Drug	Events/*N* on Placebo	OR (95% CI)^a^
**Aripiprazole**	Berman (2007) [75]	Fatigue	11/182	6/176	
	Berman (2009) [82]	Fatigue	16/176	8/172	
		Sedation	10/176	1/172	
	Marcus (2008) [76]	Fatigue	19/189	7/190	
		Somnolence	13/189	7/190	
	**Total**	Sedation-related	69/547	29/538	2.56 (1.63–4.03)
	Berman (2007) [75]	Tremor	6/182	8/176	
		Other EPS-related events	2/182	1/176	
	Berman (2009) [82]	Dyskinesia	2/176	0/172	
		Extrapyramidal disorder	2/176	0/172	
		Muscle spasms	4/176	1/172	
		Muscle twitching	3/176	3/172	
		Psychomotor activity	1/176	0/172	
		Tremor	5/176	6/172	
	Marcus (2008) [76]	Tremor	12/189	5/190	
	**Total**	EPS-related	37/547	24/538	1.54 (0.86–2.74)
	Berman (2007) [75]	Metabolic labs^b^	?	?	
	Berman (2009) [82]	Metabolic labs^c^	?	?	
	Marcus (2008) [76]	Metabolic labs^d^	?	?	
	**Total**	Metabolic labs	?	?	
	Berman (2007) [75]	Akathisia	42/182	8/176	
		Restlessness	26/182	6/176	
	Berman (2009) [82]	Akathisia	32/176	6/172	
		Restlessness	22/176	6/172	
	Marcus (2008) [76]	Akathisia	49/189	8/190	
		Restlessness	18/189	1/190	
	**Total**	Akathisia-related	189/547	35/538	7.47 (5.07–11.0)
	Berman (2007) [75]	Weight gain ≥7%	13/182	2/176	
	Berman (2009) [82]	Weight gain ≥7%	8/176	2/172	
	Marcus (2008) [76]	Weight gain ≥7%	6/189	0/190	
	**Total**	Weight gain ≥7%	27/547	4/538	5.91 (2.14–16.29)
**OFC**	Corya (2006) [25]	Asthenia	29/243	10/119	
		Somnolence	53/243	8/119	
	Shelton (2001) [27]	Asthenia	5/10	4/10	
		Somnolence^e^	6/10	5/10	
	Shelton (2005) [26]	Asthenia	30/146	25/210	
		Somnolence	25/146	27/210	
	Thase (2007) [85]	Fatigue	28/200	16/206	
		Hypersomnia	21/200	5/206	
		Sedation	19/200	7/206	
		Somnolence	35/200	11/206	
	**Total**	Sedation	240/589	109/535	2.87 (1.64–5.03)
	Corya (2006) [25]	Dyskinesia any time (AIMS)	1/227	3/113	
		Dyskinesia at last two visits (AIMS)	0/227	1/113	
		Dyskinesia at end point (AIMS)	1/227	1/113	
		Parkinsonism (SAS)	6/226	7/113	
	Shelton (2001) [27]	Parkinsonism (SAS)	0/10	3/9	
	Shelton (2005) [26]	Dyskinesia any time (AIMS)	2/140	0/197	
		Dyskinesia at last two visits (AIMS)	0/140	0/197	
		Dyskinesia at end point (AIMS)	0/140	0/197	
		Parkinsonism (SAS)	7/140	1/199	
		Tremor	17/146	8/210	
	Thase (2007) [85]	Dyskinesia any time (AIMS)	1/196	3/201	
		Dyskinesia at last two visits (AIMS)	0/195	0/201	
		Dyskinesia at end point (AIMS)	0/194	1/199	
		Parkinsonism (SAS)	5/192	2/195	
		Tremor	21/200	18/206	
	**Total**	EPS-related	61/574	48/522	0.88 (0.25–3.04)
	Corya (2006) [25]	Cholesterol high	12/213	0/103	
		Nonfasting glucose high	6/209	0/103	
		HbA1c high	10/153	1/77	
	Shelton (2001) [27]	Cholesterol high	1/10	0/10	
		Nonfasting glucose high	0/10	0/10	
	Shelton (2005) [26]	Cholesterol high	3/133	7/193	
		Nonfasting glucose high	8/131	3/192	
		Hyperglycemia	3/146	0/200	
	Thase (2007) [85]	Cholesterol high	9/189	3/194	
		Fasting glucose high	2/28	0/36	
		Nonfasting glucose high	6/168	1/170	
		HbA1c high	8/144	0/165	
		Triglycerides high	10/189	3/196	
	**Total**	Metabolic labs^f^	78/482	18/455	4.46 (2.07–9.58)
	Corya (2006) [25]	Agitation	14/243	4/119	
		Akathisia any time (Barnes)	23/227	5/109	
	Shelton (2001) [27]	Akathisia	2/10	0/10	
		Akathisia (Barnes)	3/10	2/9	
	Shelton (2005) [26]	Akathisia (Barnes)	14/138	20/196	
	Thase (2007) [85]	Akathisia (Barnes)	18/188	13/188	
	**Total**	Akathisia	74/571	44/508	1.48 (0.96–2.30)
	Corya (2006) [25]	Prolactin high	43/186	7/89	
	Shelton (2001) [27]	Prolactin high	4/9	0/7	
	Shelton (2005) [26]	Prolactin high	34/119	6/178	
	Thase (2007) [85]	Prolactin high	49/159	23/172	
	**Total**	Prolactin high^g^	130/473	36/446	4.30 (2.36–7.83)
	Corya (2006) [25]	Peripheral edema	27/243	1/119	
		Edema	19/243	1/119	
	Shelton (2001) [27]	Peripheral edema	2/10	0/10	
	Thase (2007) [85]	Peripheral edema	24/200	2/206	
		Edema	11/200	1/206	
	**Total**	Edema	83/453	5/335	13.19 (5.46–31.89)
	Corya (2006) [25]	Weight gain ≥10%	53/230	2/114	
	Shelton (2001) [27]	Weight gain ≥10%	3/10	0/10	
	Shelton (2005) [26]	Weight gain >10%	11/146	0/210	
	Thase (2007) [85]	Weight gain ≥10%	42/198	2/203	
	**Total**	Weight gain >10% or weight gain ≥10%	109/584	4/537	16.28 (7.02–37.76)
**Quetiapine**	Bauer (2009) [81]	Fatigue	46/330	5/161	
		Lethargy	7/330	2/161	
		Sedation	37/330	7/161	
		Somnolence	66/330	5/161	
	El-Khalili (2010) [83]	Fatigue	33/297	7/148	
		Hypersomnia	6/297	0/148	
		Sedation	58/297	6/148	
		Somnolence	86/297	6/148	
	McIntyre (2007) [84]	Sedation/somnolence/lethargy	25/29	14/29	
	**Total**	Sedation-related	364/656	52/338	8.36 (5.83–11.98)
	Bauer (2009) [81]	EPS-related	None	None	
	El-Khalili (2010) [83]	EPS-related	17/297	5/148	
	McIntyre (2007) [84]	EPS-related	None	None	
	**Total**	EPS-related	17/297	5/148	1.66 (0.59–4.67)
	Bauer (2009) [81]	Fasting glucose high	15/330	4/161	
		LDL cholesterol high	47/330	18/161	
		HDL cholesterol low	13/330	7/161	
		Total cholesterol high	60/330	14/161	
		Triglycerides high	40/330	5/161	
	El-Khalili (2010) [83]	Fasting glucose high	11/297	5/148	
		HbA1c high	2/297	1/148	
		HDL cholesterol low	18/297	7/148	
		LDL cholesterol high	12/297	5/148	
		Total cholesterol high	22/297	2/148	
		Triglycerides high	29/297	6/148	
	McIntyre (2007) [84]	Metabolic labs^h^	?	?	
	**Total**	Metabolic labs^i^	269/627	74/309	2.45 (1.80–3.34)
	Bauer (2009) [81]	Shift from <3 to ≥3 metabolic risk factors	27/330	16/161	
	El-Khalili (2010) [83]	Shift from <3 to ≥3 metabolic risk factors^j^	50/297	9/148	
	**Total**	Shift from <3 to ≥3 metabolic risk factors	77/627	25/309	1.57 (0.42–5.92)
	El-Khalili (2010) [83]	Akathisia	6/297	1/148	
		Restlessness	5/297	2/148	
	**Total**	Akathisia-related	11/297	3/148	1.75 (0.47–6.55)
	Bauer (2009) [81]	Elevated prolactin^k^	6/330	3/161	
	**Total**	Elevated prolactin	6/330	3/161	0.96 (0.23–3.96)
	Bauer (2009) [81]	Weight gain ≥7%	14/330	2/161	
	El-Khalili (2010) [83]	Weight gain ≥7%	13/297	3/148	
	McIntyre (2007) [84]	Weight gain ≥7%	4/18	0/14	
	**Total**	Weight gain ≥7%	31/645	5/323	2.86 (1.11–7.37)
**Risperidone**	Keitner (2009) [73]	Fatigue	0/62	2/33	
		Tired	0/62	2/33	
	Mahmoud (2007) [44]	Fatigue	5/137	0/131	
		Lethargy	1/137	3/131	
		Somnolence	7/137	2/131	
	Reeves (2008) [77]	Somnolence	2/12	1/11	
	**Total**	Sedation-related	15/211	10/175	0.88 (0.11–7.55)
	Mahmoud (2007) [44]	Dystonia	0/137	1/131	
		Tremor	1/137	1/131	
	**Total**	EPS-related	1/137	2/131	0.47 (0.04–5.29)
	Keitner (2009) [73]	Metabolic labs^l^	?	?	
	Mahmoud (2007) [44]	Metabolic labs^l^	?	?	
	Reeves (2008) [77]	Metabolic labs^l^	?	?	
	**Total**	Metabolic labs	?	?	
	Mahmoud (2007) [44]	Akathisia	1/137	0/131	
	**Total**	Akathisia-related	1/137	0/131	2.89 (0.12–71.58)
	Keitner (2009) [73]	Edema	0/62	0/33	
	Mahmoud (2007) [44]	Peripheral edema	4/137	1/131	
	Reeves (2008) [77]	Edema	0/12	0/11	
	**Total**	Edema	4/211	1/175	3.91 (0.43–35.45)
	Keitner (2009) [73]	Weight gain ≥7%	2/62	0/33	
	Mahmoud (2007) [44]	Weight gain ≥7%^m^	?	?	
	Reeves (2008) [77]	Weight gain ≥7%^m^	?	?	
	**Total**	Weight gain ≥7%	2/62	0/33	2.77 (0.13–59.38)

a Trials with no events in either study arm are not included in summary OR calculations.

b The clinical registry report indicated that statistically significant differences emerged between drug and placebo in glucose, total cholesterol, fasting LDL cholesterol, nonfasting and fasting triglycerides, and prolactin. These differences were not reported quantitatively and were described as not “clinically meaningful.”

c Median levels of change in fasting total cholesterol, triglycerides, HDL cholesterol, LDL cholesterol, and fasting plasma glucose were reported. Categorical measures (i.e., numbers of patients who had abnormal scores) were not reported. The clinical trial registry noted that there was a statistically significant but clinically nonmeaningful difference between drug and placebo on nonfasting LDL cholesterol.

d Data on metabolic parameters were reported in terms of median change, but no categorical reporting of laboratory abnormalities was provided. Differences between drug and placebo were reported as not statistically significant in terms of median change on glucose, cholesterol, and triglycerides.

e Because the total number of events in the OFC group was higher than the sample size of the group, an effect size could not be calculated, and it was thus not factored into the overall effect size estimate for sedation. Given the very small sample of the study, this makes virtually no difference in the overall effect size estimate.

f The number of participants providing data differed substantially across metabolic testing parameters. The average sample size across the metabolic testing groups provided the denominator for the pooled number of abnormal metabolic test results in each trial, with the total number of participants who experienced an abnormal metabolic testing result comprising the numerator. A participant may have experienced more than one event. Also, boundaries of abnormal tests were defined by standard Lilly reference ranges, a resource not available to our research team.

g Boundaries of abnormal tests were defined by standard Lilly reference ranges, a resource not available to our research team.

h Triglycerides and unclearly described laboratory tests were completed in this study, but the results were described only as yielding “no clinically significant differences” between groups.

i Abnormal metabolic laboratory values were defined as follows: fasting glucose ≥126 mg/dl, LDL cholesterol ≥160 mg/dl, HDL cholesterol ≤40 mg/dl, total cholesterol ≥240 mg/dl, and triglycerides ≥200 mg/dl.

j The clinical trial registry entry noted that approximately 17% of patients taking quetiapine met this criterion, compared to 6% of placebo patients. We extracted numbers of patients based on these percentages.

k Defined as >20 ng/ml for males and >30 ng/ml for females.

l These parameters were apparently not measured.

m Weight gain was provided in terms of means and standard deviations; however, no categorical measure of significant weight gain was reported.

AIMS, Abnormal Involuntary Movement Scale; Barnes, Barnes Akathisia Scale; HbA1c, glycated hemoglobin; SAS, Simpson-Angus Scale.

Adjunctive aripiprazole was frequently associated with akathisia (NNH, 4; 95% CI, 3–6) and also linked to a statistically significant elevation in the occurrence of sedation (NNH, 14; 95% CI, 8–33) and significant weight gain of ≥7% during trials (NNH, 29; 95% CI, 10–119). Adjunctive OFC was often associated with significant weight gain of >10% or ≥10% (NNH, 9; 95% CI, 5–20), sedation (NNH, 5; 95% CI, 3–12), abnormal metabolic laboratory results (NNH, 10; 95% CI, 5–29), and elevated prolactin (NNH, 6; 95% CI, 4–11). Adjunctive quetiapine had a very high rate of reported sedation (NNH, 3; 95% CI, 2–3) and was also linked to abnormal metabolic laboratory results (NNH, 6; 95% CI, 4–9) and significant weight gain of ≥7% (NNH 37; 95% CI, 12–594). Adjunctive risperidone was not associated with an increased rate of any spontaneously reported adverse events.

All four drugs resulted in statistically significant weight gain (Table 3): mean weight gain in trials of adjunctive aripiprazole, quetiapine, and risperidone was approximately 1 kg, while the average weight gain resulting from adjunctive OFC was 4.20 kg (95% CI, 3.79–4.61). OFC was associated with more weight gain than the other drugs (*Q*_B_ = 58.46, *p*<0.001), which did not differ significantly from each other (*Q*_B_ = 0.66, *p* = 0.72).

The thresholds for adverse event reporting in the included publications are shown in Table 1. Adverse events were typically listed in a table and were reported only if a certain proportion of study participants reported that event. For example, if only those adverse events reported by 5% or more of participants in either group were reported in the published journal article, we describe it in Table 1 as “≥5% in any group.” In general, little to no additional information was provided in the study publications regarding adverse events beyond that which was presented in such tables. Meta-analysis of effects on sexual functioning rating scales was not performed because of the often unclear reporting of these measures (see Table 3).

### Publication Bias

**Figure 4:**
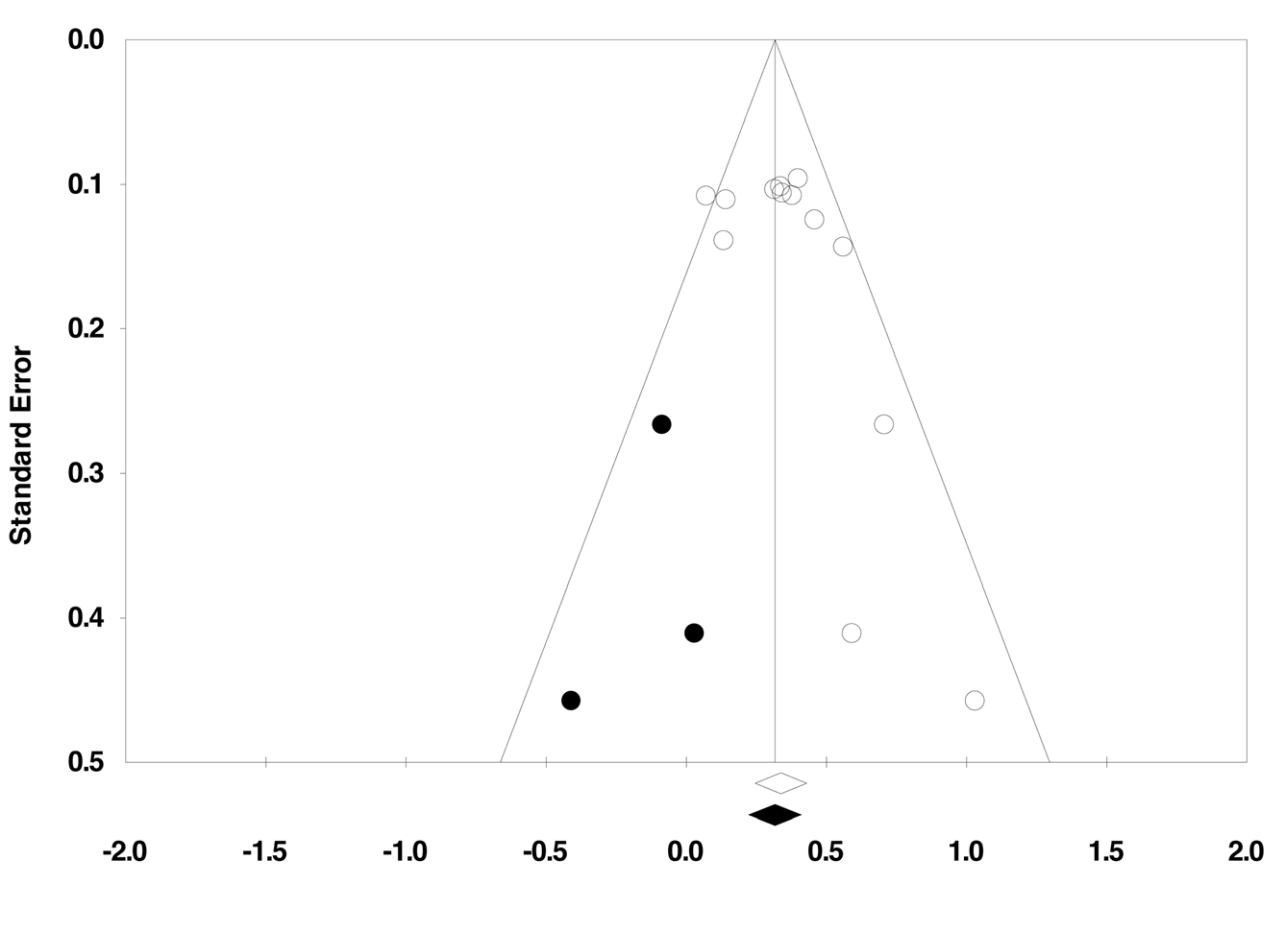
Funnel plot of trim and fill analysis. Open circles represent published studies; filled circles represent imputed unpublished studies. The overall effect size changes from 0.34 to 0.32 when including these imputed trials.

The trim and fill procedure suggested the existence of three unpublished trials, bringing the overall effect on depression measures to 0.32. A funnel plot showing the results of this analysis can be seen in Figure 4.

## Discussion

In this meta-analysis of 14 randomized trials of atypical antipsychotic medications used for the adjunctive treatment of major depressive disorder, we found that all included atypical antipsychotics were more efficacious than adjunctive placebo in terms of their effects on depressive symptom severity and remission. However, the effect sizes were small or small-to-moderate in magnitude, and OFC did not generate a statistically significant benefit on treatment response. All of the studied drugs except risperidone demonstrated substantial risk of several adverse events. Our findings have clinically important implications for comprehensively understanding the risk–benefit profiles of these adjunctive treatments for major depressive disorder.

The overall effect size on depression severity was *g* = 0.34, an effect conventionally deemed as small. In a meta-analysis of antidepressants versus placebo, Kirsch et al. found an effect size of 0.32, which they interpreted as not clinically relevant [46]. This was in line with the recommendations of the National Institute for Health and Clinical Excellence in the United Kingdom, which deemed effect sizes of *g*<0.5 as clinically insignificant, though no evidence was cited for this cutoff [47]. However, Turner et al.'s meta-analysis of antidepressants versus placebo found an effect size of 0.31 [11], which was interpreted as “measurable and significant” [48]. These differing interpretations are understandable given that Cohen noted that his original categorization of effect sizes (0.2 = small, 0.5 = medium, and 0.8 = large) was arbitrary [45]. We interpret the effect of adjunctive antipsychotic treatment on depression measures as of questionable clinical relevance. In addition, sole reliance on depression rating scales to determine treatment benefit is likely inadequate in understanding overall treatment efficacy.

The pooled difference in mean change across 11 trials was 2.69 points on the MADRS. The MADRS consists of ten items, each rated on a 0–6 scale, assessing sadness, inner tension, reduced sleep, loss of appetite, concentration, difficulty with starting daily activities, inability to feel, pessimism, and suicidal thoughts. A small difference favoring an atypical antipsychotic over placebo on the MADRS may thus reflect small differences across several dimensions, or perhaps a sizable difference on one or two dimensions combined with nil differences on other items. For instance, a pooled analysis of the two large quetiapine trials included in our meta-analysis found that quetiapine at 150 mg/d and quetiapine at 300 mg/d were superior to placebo by 2.50 and 2.85 points on the MADRS at study end point, respectively [49]. The treatment advantage in terms of the items “apparent sadness” and “reported sadness” appears to be about a third of that for the “reduced sleep” item (Figure 3b of [49]), suggesting that quetiapine's sedative effect on sleep may account for a substantial degree of the observed improvement in depression scores. Thus, improvement in overall depression rating scales should be interpreted cautiously.

Response and remission rates are often used to convey the magnitude of treatment benefit; however, these categorical measures are created arbitrarily from underlying continuous rating scale data [50]. In some circumstances these categorical measures may inflate treatment differences relative to mean change on the continuous scale [51]. While response and remission rates are potentially useful outcome measures, they should be considered only in the context of a wider set of outcome data.

With the exception of risperidone, nearly all of the included trials estimated small or minimal benefits with regards to quality of life and functional impairment. Quetiapine and OFC generated no benefit on such measures, whereas the benefits of aripiprazole were statistically significant yet quite modest. Although risperidone appeared to possess the strongest risk–benefit profile in our analyses, our findings about risperidone were based on the smallest sample size of any of the included drugs. We also have concerns about data reported in the largest risperidone trial. The published version of the study emphasizes outcomes at the end of the 6-wk trial [44]. However, in its discussion section and the trial's associated ClinicalTrials.gov registry entry [52], it is mentioned that the primary study end point was actually 4 wk; this is mentioned neither in the paper's methods section nor in the abstract. The effect size on the HAM-D is 30% smaller at the 4-wk end point relative to the 6-wk end point. Effects on the Q-LES-Q and SDS were reported only at week 6, but it seems likely that these effects would be smaller at the primary study end point. Given that this study included 69% of the total participants in risperidone trials, our pooled estimate of risperidone efficacy is therefore driven by the inclusion of post hoc analyses. Further, a previously published relapse prevention study (not included in our meta-analysis due to its study design) found no benefit for risperidone over placebo, suggesting that risperidone-related gains may be transient [53],[54].

Taken together, our findings raise significant concerns regarding the impact of these medications in improving *overall* well-being. Although improvements in quality of life or functional status commonly co-occur with improvements in depression symptom severity, this cannot automatically be assumed. One comprehensive literature review estimated only a moderate degree of correlation between these constructs [55]. It has been argued that changes on quality of life measures may lag changes on depressive symptom measures and that short-term trials may not be an appropriate setting in which to estimate changes on quality of life measures [15]. Contrary to this argument, however, four of five recently published short-term antidepressant medication trials found that benefits of medication over placebo were similar on measures of (1) quality of life or functional impairment (e.g., as measured by the Q-LES-Q and SDS) and (2) depression symptom severity (e.g., as measured by the HAM-D and MADRS) [56]–[60]. Our findings highlight the fact that reporting data only on symptom response and resolution may provide an incomplete and perhaps overly optimistic summary of a medication's overall effects on well-being [15],[16],[61]. More robust assessments of quality of life and functional impairment should be incorporated into the design of clinical trials of any putative antidepressant.

Without longer-term data on not only depression symptom severity but also quality of life and social functioning, it is difficult to assess the risk–benefit profile of these medications prescribed over the long term. None of the included trials provided data on long-term (i.e., ≥6 mo) outcomes comparing adjunctive antipsychotic medication treatment to adjunctive placebo. Our failure to find long-term outcome data is consistent with that of previous research teams [17],[62]. For example, one systematic review of long-term, two-arm parallel randomized controlled antidepressant trials initially identified 2,693 abstracts, only to ultimately include six trials [62]. This limitation is shared with other treatments; there is very little understanding of how adjunctive treatments for depression influence long-term well-being.

In addition to providing a thorough assessment of efficacy outcomes, our meta-analysis departs from the literature in a second notable way by comprehensively summarizing the available safety information on these medications. Such safety data have not been included in prior quantitative reviews, but our conclusions echo concerns raised in previous meta-analyses and a narrative review regarding potential treatment-related harms associated with use of atypical antipsychotic medication in the adjunctive treatment of depression [6]–[8]. Overall, we found that treatment was linked to several adverse events, including akathisia (aripiprazole), sedation (quetiapine, OFC, and aripiprazole), abnormal metabolic laboratory results (quetiapine and OFC), and weight gain (all four drugs, especially OFC). Measures of absolute benefit and harm (NNT and NNH) provide an intuitive metric for understanding treatment-related benefits and harms. However, these measures are dependent on baseline control group risk, which may vary substantially across clinical subgroups [63]. Thus, our findings in terms of NNT and NNH should be interpreted as estimates of effects for each drug relative to control participants who may differ from participants treated in clinical practice.

Our ability to provide an adequate safety profile of these medications was limited in two respects. First, while 11 of 14 included trials used a structured instrument to elicit adverse events, these measures were limited to assessing potential EPS- and akathisia-related events, and, in five studies, sexual functioning. No study reported using a structured checklist to elicit adverse events outside of EPS, akathisia, or sexual functioning, which is a substantial limitation given that adverse events are reported with as much as 20 times greater frequency when elicited through structured checklists versus being recorded in response to patient complaints [19],[20]. The importance of measuring adverse events systematically was demonstrated historically in the case of selective serotonin reuptake inhibitors: in registration trials, sexual dysfunction was neither systematically assessed nor found to be frequently spontaneously reported by patients [64],[65]. Further investigation indicated, however, that sexual side effects on selective serotonin reuptake inhibitors are actually quite common [19]. While the collection of adverse event data via structured checklists is a more sensitive method of collecting adverse event data, it may result in many common (mostly minor) health problems being endorsed even if they are not due to treatment, potentially leading to decreased specificity in differentiating medications from placebo [66]. To bridge the differences between the systematic and open-ended assessment of adverse events in clinical trials, some sort of hybrid method of collecting adverse event data could be performed, such as randomly assigning some participants within both the active treatment and placebo groups to complete a structured checklist while assigning others to complete an open-ended assessment of adverse events.

A second constraint on our ability to adequately summarize the drugs' safety profiles is that many adverse events were not reported in journal articles and that some of the data were incomplete or reported in a fashion that may have obscured treatment-related harms. We agree with the Cochrane reviewers that “data on side effects were often very poorly described” [9]. Conceptually similar events such as sedation, fatigue, and somnolence were sometimes reported separately, often with no attempt to pool them together. This is in direct contradiction to FDA guidance, which states that events that “represent the same phenomenon (e.g., somnolence, sedation, drowsiness) should ordinarily be grouped together as a single adverse reaction to avoid diluting or obscuring the true effect” [67].

Given the notable side effect profiles of the studied drugs, it is likely that the double-blind was significantly compromised; however, none of the included trials tested the integrity of blinding. For example, patients who rapidly gained weight in an OFC trial, who complained of akathisia in an aripiprazole trial, or who reported sedation in a quetiapine trial would likely cue the awareness of study personnel that they were assigned to the active drug condition. Assuming that proper informed consent was obtained, participants were also likely to accurately guess their treatment assignment based on side effect cues [68],[69]. This could have led to inflated efficacy ratings by clinical raters and participants [70],[71]. The lack of protocols assessing the integrity of the double-blind in the trials included in our meta-analysis is consistent with the wider clinical trials literature [72]. The potential for unblinding to cause inflated efficacy ratings among clinical raters could be substantially limited if efficacy outcomes were assessed by different personnel than those who assessed adverse events [70]. Yet the use of separate raters to assess efficacy and safety outcomes was reported in only one trial [73].

The FDA statistical reviewer for aripiprazole [74] wrote regarding Berman et al. [75] that “the medical reviewer is concerned about the considerable number of protocol violations in the study primarily due to usage of opiates/barbiturates” [74]. Regarding the Marcus et al. [76] trial, the FDA reviewer wrote that the difference between groups in the number of participants who used prohibited medications was “huge” [74], with nine patients in the placebo group doing so compared to 24 in the aripiprazole group. The reviewer thus performed a separate analysis, excluding patients in the two trials who violated the study protocol, the results of which indicated a minimal, non-statistically significant effect of aripiprazole on functional status. In the journal articles, this potentially important issue is not mentioned. The FDA reviewer reported results only from reanalysis of the MADRS and SDS, so it is unknown to what extent these protocol violations may have impacted results on other outcome measures [74].

Our results differ somewhat from those of Nelson and Papakostas, whose meta-analysis concluded that augmentation with atypical antipsychotics was effective and, further, that “this body of evidence is considerably larger than that for any other augmentation strategy in the treatment of major depressive disorder” [6]. There are seven differences in our analyses that provide reasons why we reached different conclusions. The greatest divergence in our results was regarding OFC, for which we found a lower OR favoring OFC for remission (1.42) than did Nelson and Papakostas (1.83). In this first instance, Nelson and Papakostas utilized whatever definition of remission was provided by the authors of each study, whereas we used a more restrictive definition. Three OFC trials defined remission as achieving a MADRS score of ≤8 at two consecutive visits—even if patients relapsed during the trial [25]–[27]. We found that after meeting criteria for remission, OFC-treated participants were more likely to relapse than placebo-treated participants; this contributed to our finding a less favorable result for OFC in terms of remission. Second, we extracted data from all comparison groups that received adjunctive placebo treatment, whereas Nelson and Papakostas excluded one comparison group from each of two OFC trials [25],[26]. Third, Nelson and Papakostas estimated a significant treatment effect for OFC on response, whereas we did not. This difference seems due to a combination of our inclusion of all adjunctive placebo comparison groups and our use of random effects analysis as opposed to their use of a fixed effects model [38]. Our fourth point of difference was that Nelson and Papakostas included data from two conference presentations on quetiapine that showed positive findings; we were unable to obtain data from these authors despite three emailed requests over a span of 4 wk. Additionally, we attempted to contact one author via phone; the attempt did not result in the release of any data. Nonetheless, the pooled ORs generated in our analyses for quetiapine in terms of response (1.53) and remission (1.79) were quite similar to those published in Nelson and Papakostas's meta-analysis (1.60 and 1.89, respectively). Our fifth difference was the use of different definitions of remission in one risperidone trial [73], and the sixth difference was Nelson and Papakostas's inclusion of data from one small risperidone trial from which we were unable to extract remission data [77], leading to our finding a slightly lower rate of remission (OR of 2.37 versus 2.63). Lastly, and most importantly, the primary point of difference is that our analysis provides a more comprehensive appraisal of treatment efficacy and safety, which, as discussed above, presents a more accurate assessment of the comparative risks and benefits of treatment.

Our review adds to the Cochrane review on this topic [9] by filling in three important data gaps: (1) unpublished data from the FDA and clinical trial registry reports, (2) data on functioning and quality of life outcomes, and (3) data on metabolic laboratory parameters. Thus, our dataset contained more outcomes and often provided a more comprehensive assessment of included outcomes than the Cochrane review. For instance, the Cochrane review included data from one trial that reported data on clinically significant weight gain for patients on OFC, whereas we included data on both mean weight changes and binary measures of clinically significant weight gain from four such trials. We included laboratory data for several metabolic parameters for both quetiapine and OFC. Despite some differences in methodology, we agree with the Cochrane review that the evidence supporting the use of adjunctive atypical antipsychotics for depression is modest.

Several methodological issues also bear mention. First, while all trials were described as randomized, double-blind trials, only three trials clearly described adequate sequence generation procedures; in the remaining studies, such procedures were unclear. A lack of appropriate randomization or differences in the taste, smell, or appearance of the medication and placebo may allow study personnel and/or participants to guess their treatment assignment. As purportedly double-blind trials with unclear or inadequate randomization are associated with larger effects than trials in which adequate randomization is clearly described, this leads to the possibility that the current set of efficacy ratings were inflated to an unknown extent [21],[78]. Second, the design of some of the included trials may have compromised their validity. In each of the aripiprazole trials, patients were treated with an antidepressant plus adjunctive placebo for 8 wk; at that point, those who showed a treatment response were eliminated from the study, and the remaining patients were assigned to either remain on the same treatment or receive adjunctive aripiprazole in place of adjunctive placebo. Thus, all patients taking placebo during the randomized trial had clearly demonstrated poor response to placebo treatment and were likely predisposed to perform poorly during the randomized portion of the trial, thereby possibly inflating the estimated efficacy of the study drug [79].

In any systematic review, publication bias is a potentially serious problem [10],[80]. To incorporate as much data as possible, we conducted a thorough literature search and included unpublished data. We did not uncover the existence of any additional unpublished negative trials in our search, but this does not mean that such trials do not exist. Given the small number of trials for each drug in our study, we lacked statistical power to conduct a formal analysis of publication bias for each drug. However, when pooling across drugs, we detected that publication bias may have slightly enhanced the overall effect size on depression measures. Our results likely represent an upper boundary for the efficacy of these compounds (as demonstrated in prior meta-analyses), assuming that relevant unpublished data are more negative than positive in terms of efficacy [10],[11].

We are aware of no trials that have directly compared adjunctive atypical antipsychotic medication treatment to other adjunctive treatments such as psychotherapy or lithium, or to other treatment strategies such as switching the antidepressant medication initially used for treatment. Further study may answer critical outstanding questions regarding the safety profiles and longer-term outcomes associated with these medications. Taken together, our meta-analysis found evidence of (1) some improvement in clinician-assessed depressive symptoms, (2) little evidence of substantial benefit in overall well-being, and (3) abundant evidence of potential treatment-related harm. Our comprehensive evaluation of safety and both relative and absolute efficacy provides critical insight that may be useful for clinicians attempting to thoroughly understand the risk–benefit profiles of these adjunctive treatments for major depressive disorder.

## Supporting Information

Text S1 **PRISMA 2009 checklist.**
(DOC)

## Abbreviations

EPS: extrapyramidal symptoms
FDA: US Food and Drug Administration
HAM-D: Hamilton Depression Rating Scale
HDL: high-density lipoprotein
LDL: low-density lipoprotein
MADRS: Montgomery–Asberg Depression Rating Scale
NDA: New Drug Application
NNH: number needed to harm
NNT: number needed to treat
OFC: olanzapine/fluoxetine combination
OR: odds ratio
Q-LES-Q: Quality of Life Enjoyment and Satisfaction Questionnaire
SDS: Sheehan Disability Scale
SF-36. Short Form 36 Health Survey
